# The value of ^99m^Tc-MAA SPECT/CT for lung shunt estimation in ^90^Y radioembolization: a phantom and patient study

**DOI:** 10.1186/s13550-018-0402-8

**Published:** 2018-06-15

**Authors:** Jonathan D. Allred, Jeremy Niedbala, Justin K. Mikell, Dawn Owen, Kirk A. Frey, Yuni K. Dewaraja

**Affiliations:** 1 Radiotherapy Physics, Nottingham University Hospitals NHS Trust, Nottingham, UK; 2 Department of Radiology, University of Michigan, 1301 Catherine, 2276 Med Sci I/SPC 5610, Ann Arbor, MI 48109 USA; 3 Department of Radiation Oncology, University of Michigan, Ann Arbor, USA

**Keywords:** ^90^Y PET/CT, Transarterial radioembolization (TARE), Lung shunt, ^99m^Tc-MAA SPECT/CT

## Abstract

**Background:**

A major toxicity concern in radioembolization therapy of hepatic malignancies is radiation-induced pneumonitis and sclerosis due to hepatopulmonary shunting of ^90^Y microspheres. Currently, ^99m^Tc macroaggregated albumin (^99m^Tc-MAA) imaging is used to estimate the lung shunt fraction (LSF) prior to treatment. The aim of this study was to evaluate the accuracy/precision of LSF estimated from ^99m^Tc planar and SPECT/CT phantom imaging, and within this context, to compare the corresponding LSF and lung-absorbed dose values from ^99m^Tc-MAA patient studies. Additionally, LSFs from pre- and post-therapy imaging were compared.

**Results:**

A liver/lung torso phantom filled with ^99m^Tc to achieve three lung shunt values was scanned by planar and SPECT/CT imaging with repeat acquisitions to assess accuracy and precision. To facilitate processing of patient data, a workflow that relies on SPECT and CT-based auto-contouring to define liver and lung volumes for the LSF calculation was implemented. Planar imaging-based LSF estimates for 40 patients, obtained from their medical records, were retrospectively compared with SPECT/CT imaging-based calculations with attenuation and scatter correction. Additionally, in a subset of 20 patients, the pre-therapy estimates were compared with ^90^Y PET/CT-based measurements.

In the phantom study, improved accuracy in LSF estimation was achieved using SPECT/CT with attenuation and scatter correction (within 13% of the true value) compared with planar imaging (up to 44% overestimation). The results in patients showed a similar trend with planar imaging significantly overestimating LSF compared to SPECT/CT. There was no correlation between lung shunt estimates and the delay between ^99m^Tc-MAA administration and scanning, but off-target extra hepatic uptake tended to be more likely in patients with a longer delay. The mean lung absorbed dose predictions for the 28 patients who underwent therapy was 9.3 Gy (range 1.3–29.4) for planar imaging and 3.2 Gy (range 0.4–13.4) for SPECT/CT. For the patients with post-therapy imaging, the mean LSF from ^90^Y PET/CT was 1.0%, (range 0.3–2.8). This value was not significantly different from the mean LSF estimate from ^99m^Tc-MAA SPECT/CT (mean 1.0%, range 0.4–1.6; *p* = 0.968), but was significantly lower than the mean LSF estimate based on planar imaging (mean 4.1%, range 1.2–15.0; *p* = 0.0002).

**Conclusions:**

The improved accuracy demonstrated by the phantom study, agreement with ^90^Y PET/CT in patient studies, and the practicality of using auto-contouring for liver/lung definition suggests that ^99m^Tc-MAA SPECT/CT with scatter and attenuation corrections should be used for lung shunt estimation prior to radioembolization.

## Background

Transarterial radioembolization (TARE) with ^90^Y microspheres is an established treatment for unresectable hepatocellular carcinoma (HCC) and for liver metastases, with promising clinical results [1, 2]. In TARE, radioactive microspheres (glass or resin) are preferentially delivered and permanently implanted into hepatic tumors by exploiting the unique dual vascular anatomy of the liver. A major toxicity concern in TARE is radiation-induced pneumonitis and sclerosis due to hepatopulmonary shunting of ^90^Y microspheres. Prior to treatment, lung shunting is evaluated by surrogate imaging with ^99m^Tc-macroaggregated albumin particles (^99m^Tc-MAA) that is assumed to mimic the distribution of the microspheres. For glass microspheres, the manufacturer’s recommendation (https://www.btg-im.com/BTG/media/TheraSphere-Documents/PDF/TheraSphere-Package-Insert_USA_Rev-14.pdf) for individualization of the administered activity based on lung shunt uses an upper limit of 30 Gy for the absorbed dose to the lung relying on limited clinical observations [3, 4].

Currently, in standard clinical practice, the lung shunt fraction (LSF) is typically estimated by ^99m^Tc-MAA planar gamma camera imaging performed without accounting for attenuation or scatter effects. Because of differences in tissue densities of the lung and liver, the LSF will be overestimated when attenuation correction is not performed. Additionally, 2D imaging does not allow for accurate delineation of lung and liver regions and cannot be used to estimate the lung volume or mass for dosimetry. Although ^99m^Tc-MAA SPECT/CT is performed at many centers, for visual assessment of extra-hepatic deposition, it is not routinely used for the LSF calculation. With iterative reconstruction, image degrading physical factors such as attenuation and scatter can be accurately modeled in SPECT/CT, and tomographic 3D imaging facilitates accurate delineation of lung and liver volumes. There have been past studies that report significant overestimation in planar imaging-based LSFs compared with SPECT/CT-based estimates as well as relatively good agreement in patients undergoing Y-90 TARE [5–7]. However, these studies did not include comparison with estimates from post-therapy imaging. Additionally, only one of these studies [7] included phantom measurements to access the accuracy and precision of the imaging methodology used. Because post-therapy imaging is not part of manufacturer recommendations for TARE and is not always performed, the agreement between ^99m^Tc-MAA-based lung shunt estimates and post-therapy imaging-based estimates has not been well studied. Additionally, post-therapy ^90^Y imaging based on either bremsstrahlung SPECT/CT or ^90^Y PET/CT is challenging, especially in low uptake regions such as the lung. The use of time-of-flight information in the case of PET [8] and model/Monte Carlo-based scatter correction in the case SPECT [9–11] has led to improved Y-90 imaging, but these methods are not always available with clinic systems. Apart from the imaging methodology, another factor that can impact ^99m^Tc-MAA-based lung shunt estimation is the apparent in vivo breakdown of ^99m^Tc-MAA into smaller aggregates and to pertechnetate over time [12, 13].

Since February 2017, at our institution, in addition to planar ^99m^Tc-MAA imaging, SPECT/CT is performed with two bed positions used when the entire lung and liver are not encompassed within a single field-of-view (FOV). Thus, imaging data was available to perform SPECT/CT-based LSF calculation for comparison with the planar imaging-based calculation still used in the clinic. A workflow that uses automatic contouring tools was implemented to facilitate the process of SPECT/CT-based LSF estimation in the current study and with the goal of future clinical implementation. Experimental measurements with a liver/lung torso phantom were designed to mimic typical LSF values and imaging conditions in patients. Phantom results that establish accuracy/precision of planar and SPECT/CT-based LSF estimation used in our clinic are presented together with a retrospective comparison of patient LSF and lung absorbed dose results corresponding to the two modalities. The impact the time delay from ^99m^Tc-MAA injection to scanning has on LSF as well as on extra hepatic uptake in general is also evaluated. Additionally, some of the patients were enrolled in an ongoing research study where post-therapy ^90^Y PET/CT imaging was performed. Thus, for a subset a comparison of pre-therapy vs. post-therapy imaging-based LSF values was also performed.

## Methods

### Phantom study

A ^99m^Tc phantom study was conducted to assess accuracy/precision of lung shunt measurements from planar and SPECT/CT imaging. The phantom used was the liver/lung anatomical phantom (Data Spectrum Corporation, Durham, NC, USA) consisting of a spine insert, fillable lungs, and fillable liver modified to include two “lesions.” The activity concentrations used to fill the compartments are given in Table 1. Initially, the lungs consisting of Styrofoam beads were filled with non-radioactive water to simulate a 0% lung shunt. Next, the lungs were filled with ^99m^Tc mixed with water to simulate a 6.9% lung shunt. Subsequently, the liver activity was increased (while keeping the lung activity at the same level) to simulate a 3.6% lung shunt. This range covers clinically realistic LSF values as evident from the SPECT/CT patient data presented in the “Results” section. The maximum total activity in the phantom was 195 MBq, corresponding to the 3.6% lung shunt.

**Table 1:** Injected ^99m^Tc activity concentrations to simulate the three different lung shunt levels

	Activity concentration (kBq/mL)		
	0% LSF	3.6% LSF	6.9% LSF
Left lung	0	3	3
Right lung	0	3	3
Normal liver (liver minus lesions)	65	140	65
Spherical lesion	390	390	390
Ellipsoid lesion	390	390	390

### Patient studies

This was a retrospective study of 40 patients who had undergone ^99m^Tc-MAA imaging for lung shunt assessment prior to radioembolization with ^90^Y glass microspheres (Therasphere; BTG International Ltd., Ottawa, Canada) at the University of Michigan Medical Center. The patient population consisted of intrahepatic metastases from hepatocellular carcinoma (16), neuroendocrine cancer (4), cholangiocarcinoma (4), colorectal carcinoma (6), colon cancer (3), melanoma (3), rectal cancer (1), carcinoid (1), and adrenal cancer (2). The routine pre-therapy clinical protocol consists of administration of 185–222 MBq of ^99m^Tc-MAA followed by planar gamma camera imaging as soon after the procedure as possible. Additionally, either one or two SPECT/CTs (depending on whether the patient’s liver and entire lungs could be encapsulated in the SPECT FOV) is performed to assess extra hepatic deposition. At our institution, reconstitution of ^99m^Tc-MAA kits follows manufacturer guidelines (Jubilant DraxImage), and at the end of synthesis, elution testing is performed daily with thin layer chromatography to evaluate the radiochemical purity, with an average of 97% purity for routine production in our facility. Reconstituted kits are used within 6 h, and radiochemical purity remains stable prior to injection.

Twenty-eight of the 40 patients went on to receive ^90^Y TARE. Twenty of these patients had post-therapy ^90^Y PET/CT imaging as part of an ongoing research study, and this data was also available. Approval by the University of Michigan Institutional Review Board (IRB) was obtained to retrospectively access relevant patient data and information. Informed consent was obtained for post-therapy imaging performed for research purposes.

### Image acquisition and reconstruction

Planar and SPECT/CT imaging for both the phantom and patients were performed on Siemens Symbia systems (Intevo or T series) equipped with low-energy high-resolution (LEHR) collimators. The standard clinic acquisition protocol was used. The acquisition window was set at 15% with an adjacent 15% low-energy scatter window for SPECT. For SPECT, a 128 × 128 matrix, 60 views/head, non-circular continuous orbit was used. The patient acquisition time was 70 s each for the two (chest and abdomen) anterior/posterior planar scans and 10 s for each SPECT projection (total scan time 10 min). The CT for patients was performed in low-dose mode (130 kVp; 80 mAs) during free breathing. The phantom acquisition times were chosen to mimic count levels typical in patient imaging: 500,000 counts on the anterior planar view and 6 million total counts for the SPECT projections. The planar and SPECT/CT phantom acquisitions at each LSF were repeated three times without re-positioning to assess precision (repeatability).

Phantom and patient SPECT data were reconstructed using eight iterations four subsets of OS-EM (Siemens Flash 3D) with and without energy window-based scatter correction (SC) and CT-based attenuation correction (AC). Collimator-detector response modeling and an 8.4 mm Gaussian post-filter were used in all reconstructions.

### Lung shunt and dosimetry calculations

In the phantom study, for the planar imaging-based calculation, the liver region of interest (ROI) was defined on the anterior view and lung ROI on the posterior view by a nuclear medicine technologist following the same procedure used in patient studies. We use this approach for ROI definition because most of the liver is closer to the anterior body wall while using posterior view for lung avoids attenuation in the heart. For comparison, the phantom LSFs were also calculated using the geometric mean of liver and lung counts as this approach is used at some centers [7]. For the SPECT/CT-based calculation, liver and lung volumes were defined on the co-registered CT. LSF was calculated as:

1 $$ \mathrm{LSF}=100\times \frac{\mathrm{Counts}\ \mathrm{in}\ \mathrm{the}\ \mathrm{lung}}{\mathrm{Counts}\ \mathrm{in}\ \mathrm{the}\ \mathrm{lung}+\mathrm{counts}\ \mathrm{in}\ \mathrm{the}\ \mathrm{liver}} $$

For the patients, the planar LSFs were obtained from medical records without alteration (except in one case where the technologist had made a mistake in the calculation). These had been calculated using Eq. 1 and counts obtained by manually segmenting the liver ROI on the anterior view and lung ROI on the posterior view by nuclear medicine technologists.

**Fig. 1:**
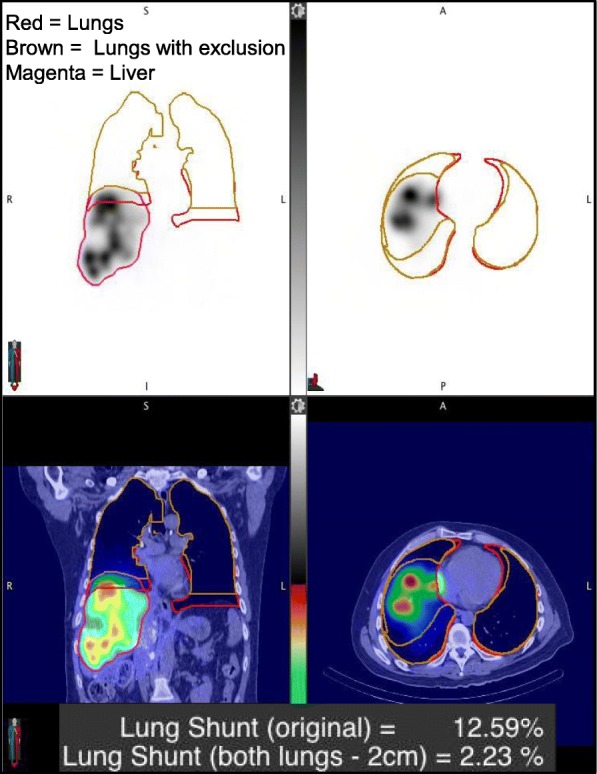
A coronal and transverse slice of a patient ^99m^Tc-MAA SPECT/CT processed through the workflow for auto contouring and LSF calculation. The CT-threshold-based lung contour with and without the 2 cm exclusion region is shown as well as the SPECT threshold-based liver contour. The lung shunt (original) refers to the value calculated without the exclusion region

To determine the patient LSFs from SPECT/CT, a semi-automatic workflow was created in MIM software (MIM Software Inc.; Beachwood, Ohio). If two SPECT/CT scans were necessary, the liver volume of interest (VOI) was defined with SPECT-based thresholding on the scan that encompassed the entire liver, and the lung VOI was defined by CT-based thresholding on the scan that encompassed the entire lung. If only one SPECT/CT scan was necessary, then the liver VOI and lung VOI were defined with the same thresholding methods, but on the singular SPECT/CT image set. To determine the liver VOI, the SPECT threshold level was selected based on visual agreement between the resulting contour and the liver outline visible on the fused CT. To automate the process, multiple liver contours corresponding to pre-set threshold levels (0.5, 1, 2, 5, and 10% of max counts in field-of-view) were made available in the workflow with the option to choose one of these contours or define a new contour. In order to determine the lung VOI, a Whole Body VOI was generated automatically through a command that detects the largest contiguous non-background region of the image by thresholding the CT image intensities and then applying a series of morphological operations. The lung VOI was automatically generated by setting an upper Hounsfield unit (HU) range lock of − 150 to this Whole Body VOI. This VOI was split into a left and right section and further cleaned using volume thresholds to remove areas in the mediastinum that may have been falsely included in the lung VOI. In order to account for the motion of the liver smearing activity into the lungs during a free breathing SPECT scan, the most inferior 2 cm of the left and right lung was automatically subtracted from the lung VOIs (Fig. 1) as proposed in the past [5, 6]. Based on the count density in the rest of the lung (the lung VOI that excludes the most inferior 2 cm) and the total lung volume from the CT, the total lung counts were estimated and the LSF was calculated within the workflow using Eq. 1.

For patients that underwent the therapy, the predicted absorbed dose to the lungs was calculated based on the MIRD formulism [14]:

2 $$ \mathrm{Lung}\ \mathrm{dose}\ \left(\mathrm{Gy}\right)=49.38\times \frac{\mathrm{Total}\ \mathrm{activity}\ \mathrm{administered}\ \left(\mathrm{GBq}\right)}{\mathrm{Lung}\ \mathrm{mass}\left(\mathrm{kg}\right)}\times \mathrm{LSF} $$

For the planar scans, the calculation was performed assuming a standard lung mass of 1 kg. For SPECT/CT, lung mass was determined by multiplying the CT-based lung volume (obtained automatically within the abovementioned workflow) by the lung density, assumed to be 0.3 kg/l [15].

### Post-therapy ^90^Y PET/CT imaging

Twenty of the patients were part of an ongoing research study where they underwent ^90^Y PET/CT imaging within a few hours of the TARE procedure. The 30-min PET/CT scan performed on a Siemens Biograph mCT with continuous bed motion was centered on the liver and included either full or partial lung (36 cm axial FOV). The CT was performed in a low-dose mode (120 kVp; 80 mAs) during free breathing. The time-of-flight PET reconstruction employed 1 iteration, 21 subsets of OSEM, and included resolution recovery and a 5-mm Gaussian post filter. These parameters were chosen based on a previous phantom evaluation of contrast, quantification, and noise [16]. The LSF based on ^90^Y PET/CT was also determined using the previously mentioned workflow. When the entire lung was not within the PET FOV (in 10/20 patients), it was necessary to approximate the total lung counts based on the count density in the part of the lung that was within the FOV and the lung volume from the CT of the ^99m^Tc-MAA SPECT/CT.

### Impact of time delay on lung shunt and extra-hepatic uptake

To evaluate the impact of time delay on LSF and extra-hepatic uptake, the elapsed time between ^99m^Tc-MAA injection and imaging was obtained from the medical records. An image review of ^99m^Tc-MAA planar head and neck images and thorax-upper abdominal SPECT/CT of each patient was performed by a Nuclear Medicine Physician (KF) to evaluate extra-hepatic uptake. Studies were rated as having none, minimal, or definitive extra-hepatic activity.

### Statistical analysis

SAS software (v9.4) was used for statistical analysis. Paired *t* tests were used to compare mean LSFs between the different imaging methods. *P* < 0.05 was considered statistically significant. Correlation between estimates from planar and SPECT/CT and between LSFs and time delay from ^99m^Tc-MAA injection to imaging was assessed using Pearson’s correlation coefficient (*R*).

## Results

### Phantom study

**Fig. 2:**
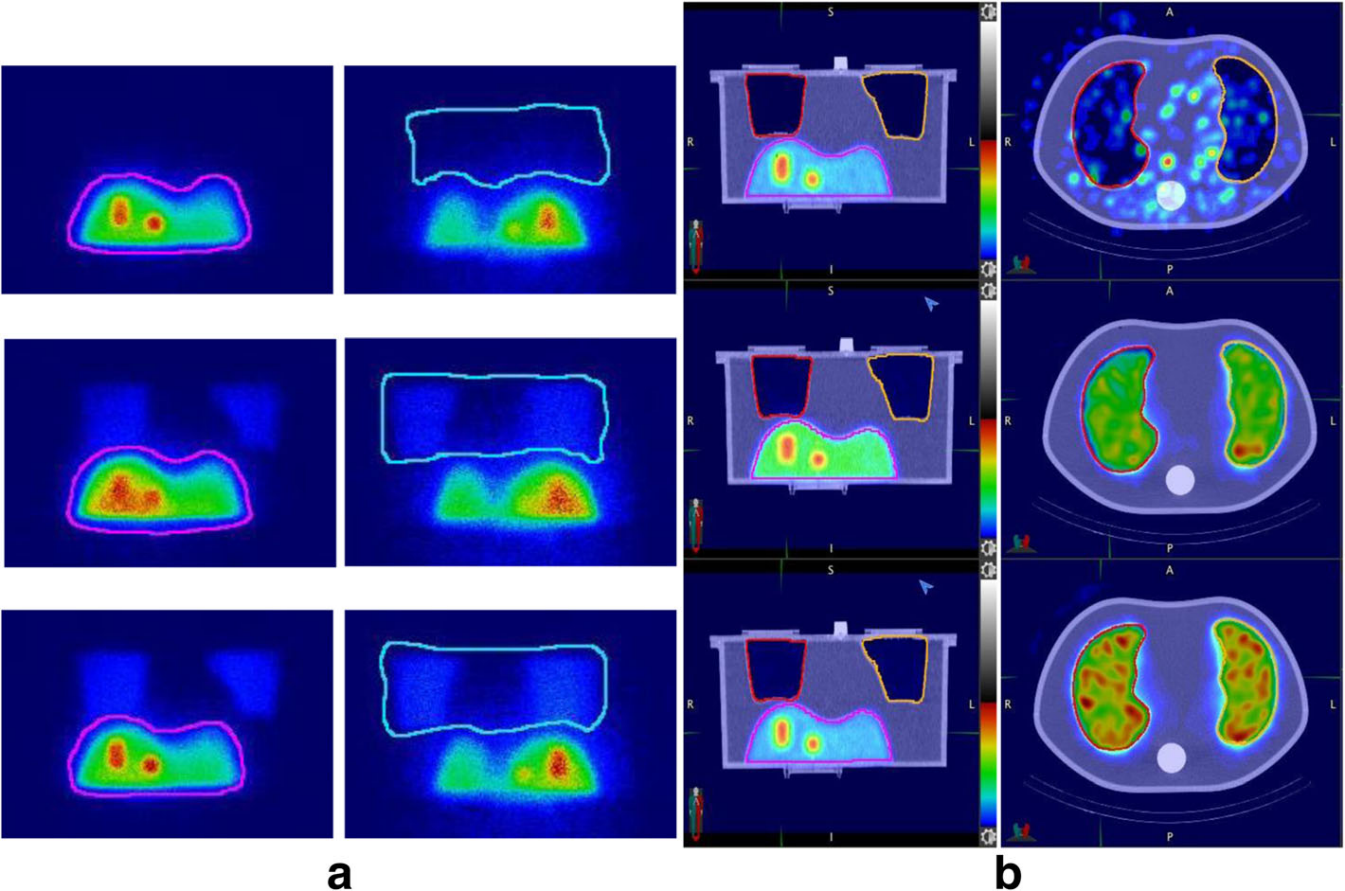
Phantom images corresponding to the different true lung shunt values 0, 3.6, and 6.9% showing **a** anterior/posterior views for planar imaging and **b** coronal and axial slices for SPECT/CT

Phantom planar and SPECT/CT images corresponding to the three different LSFs are shown in Fig. 2. Table 2 presents the mean estimated LSFs at each true lung shunt level along with the associated standard deviation from the three repeat acquisitions. LSFs estimated using the anterior liver and the posterior lung performed better than the geometric mean method. LSFs estimated from SPECT/CT with AC only, and AC and SC performed better than estimates from SPECT/CT without corrections and planar imaging.

**Table 2:** Mean lung shunt fraction (with standard deviation in parenthesis) corresponding to planar and SPECT/CT scans of the phantom

	LSF		
Actual	0.0%	3.6%	6.9%
Planar (anterior liver, posterior lung)	1.67% (0.01%)	5.12% (0.03%)	8.21% (0.05%)
Planar (geometric mean)	2.46% (0.02%)	8.17% (0.03%)	13.39% (0.02%)
SPECT/CT without corrections	1.28% (0.01%)	7.15% (0.02%)	12.13% (0.03%)
SPECT/CT with AC	0.67% (0.01%)	3.98% (0.02%)	6.83% (0.03%)
SPECT/CT with AC and SC	0.10% (0.07%)	3.10% (0.01%)	6.22% (0.03%)

### Patient studies

#### Lung shunt from ^99m^Tc-MAA imaging

**Fig. 3:**
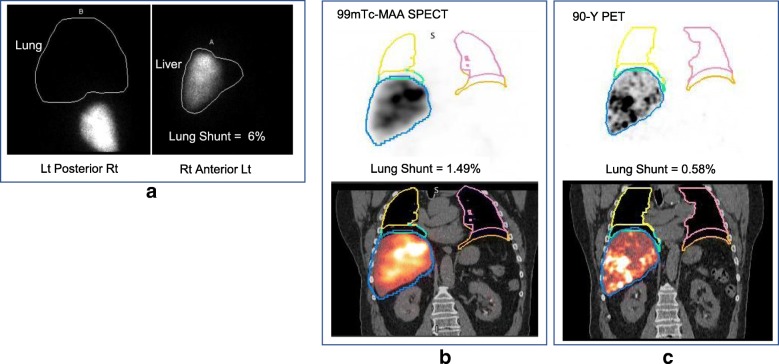
**a** Posterior, anterior ^99m^Tc-MAA planar images showing manually defined liver/lung regions for LSF calculation in the clinic. A coronal **b** ^99m^Tc-MAA SPECT/CT slice and **c** ^90^Y PET/CT slice for the same patient processed through the workflow for LSF calculation

**Fig. 4:**
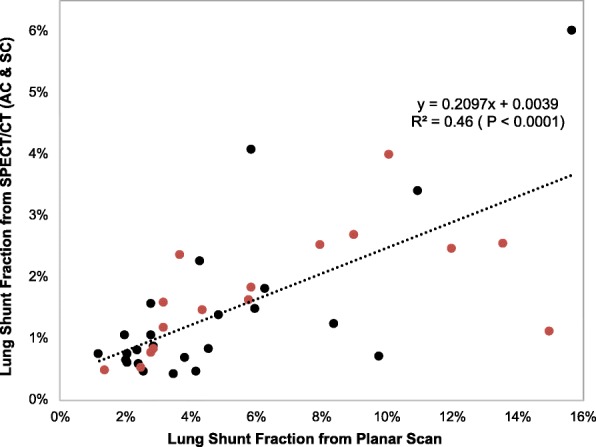
Correlation between planar and SPECT/CT derived lung shunt values for all patients (*N* = 40). Red symbols correspond to HCC patients, and black symbols correspond to patients with liver metastases

The workflow enabled automatic determination of SPECT/CT-based LSFs with only minimal user intervention to select one of the pre-set threshold levels to best define the liver contour (no contours were defined manually). The selected levels (number of patients in parenthesis) were 0.5% (2), 1% (7), 2% (20), or 5% (11). For an example patient, the lung and liver contours used in the LSF calculation corresponding to the different imaging modalities are presented in Fig. 3. The LSFs for all patients with the different imaging modalities and with and without 2-cm exclusion region are presented in Table 3. With the exclusion region, there was a substantial decrease in the LSF and all SPECT/CT and PET/CT results presented in the rest of the paper are with this correction used to mitigate motion artifacts. Results of Table 3 show that planar imaging substantially overestimates the LSF compared with SPECT/CT with AC only and AC and SC. The mean LSF from SPECT/CT (AC and SC) is 1.5% (range from 0.4 to 6.0%), and this value is statistically significantly different from LSF from planar imaging (mean 5.4%, range from 1.2 to 15.7%; *p* < 0.0001). Figure 4 shows the correlation between planar and SPECT/CT-based LSFs.

**Table 3:** Patient LSFs corresponding to the different imaging modalities and with and without the 2 cm lung exclusion area

Patient	LSF from Planar (%)	LSF from SPECT/CT with 2 cm exclusion (%)			LSF from SPECT/CT without 2 cm exclusion (%)		LSF from PET/CT with 2 cm exclusion (%)	LSF from PET/CT without 2 cm exclusion (%)
		No AC or SC	AC	AC + SC	No AC or SC	AC + SC	AC + SC	AC + SC
1	2.5	2.0	1.4	0.6	4.7	2.4	1.1	4.5
2	15.0	3.2	2.2	1.1	6.4	3.5	2.7	15.5
3	6.3	3.1	2.1	1.8	3.5	1.9		
4	10.1	7.2	5.0	4.0	11.7	7.5		
5	8.4	3.9	1.6	1.2	6.1	3.1	0.5	1.0
6	12.0	5.0	2.7	1.7	6.2	3.9		
7	3.7	4.7	2.9	2.3	5.0	2.4		
8	3.2	2.6	1.9	1.6	6.3	4.7	0.9	2.7
9	2.9	2.4	1.7	0.8	8.4	4.7	1.3	8.8
10	2.6	1.2	0.9	0.4	3.6	2.3	0.6	4.1
11	2.8	2.3	1.4	0.8	4.1	1.6		
12	2.5	2.7	1.6	0.5	5.6	2.0		
13	4.6	2.2	1.4	0.8	3.9	2.1		
14	3.8	1.3	1.1	0.7	2.6	1.6	0.4	1.4
15	1.4	1.1	0.9	0.5	3.3	2.0	0.9	4.4
16	9.8	1.3	1.1	0.7	2.9	2.0		
17	5.9	4.2	2.7	1.8	6.3	3.3		
18	9.0	7.2	4.1	2.7	13.4	7.2		
19	4.3	3.6	2.5	2.2	17.9	12.6		
20	3.2	1.9	1.4	1.2	2.7	1.8		
21	11.0	6.0	4.0	3.4	7.1	4.0		
22	4.9	3.1	2.1	1.4	3.6	1.5	0.3	0.3
23	2.1	1.4	0.3	0.7	2.1	1.3	0.4	2.2
24	2.4	1.7	1.4	0.8	5.5	4.4		
25	15.7	10.6	7.5	6.0	13.8	8.7		
26	2.0	3.1	2.2	1.0	6.0	2.6	1.5	3.0
27	8.0	3.5	3.2	2.5	3.8	2.5		
28	2.1	2.0	1.2	0.6	3.6	1.7		
29	5.9	5.6	4.1	4.1	13.1	9.1		
30	2.1	2.8	1.7	0.6	7.0	2.5		
31	2.8	2.4	2.1	1.6	14.1	9.6	1.0	4.7
32	13.6	5.5	3.6	2.5	7.5	4.3		
33	4.2	1.3	0.9	0.4	2.0	1.0	0.5	1.0
34	6.0	3.0	2.0	1.5	7.5	5.0	0.6	3.1
35	5.8	4.2	2.8	1.6	5.8	3.2	1.5	3.7
36	2.9	2.6	1.9	0.8	5.9	3.3	1.2	2.4
37	2.8	2.4	1.6	1.0	2.4	1.1	0.4	0.4
38	1.2	1.6	1.3	0.7	3.9	3.0	0.4	8.9
39	3.5	1.3	0.9	0.4	2.7	1.3	0.6	1.9
40	4.4	3.8	2.6	1.4	8.7	5.4	2.8	4.4

#### Lung absorbed dose predictions from ^99m^Tc-MAA imaging

**Fig. 5:**
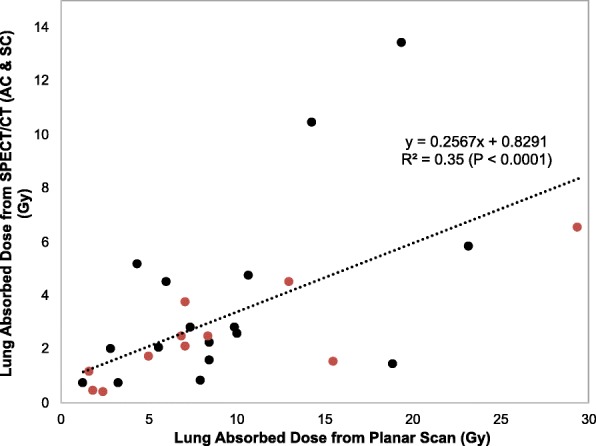
Correlation between planar and SPECT/CT derived lung absorbed dose values for patients who underwent therapy (*N* = 28). Red symbols correspond to HCC patients and black symbols correspond to patients with liver metastases

The average lung mass in this patient population, determined from the CT volume assuming a lung density of 0.3 kg/l, was 0.87 kg (range from 0.45 to 1.43). These patient-specific lung mass values were used for the SPECT/CT-based lung absorbed dose calculation while a 1-kg mass was assumed for the planar calculation. The lung doses corresponding to the planar and SPECT/CT imaging-based calculations are plotted in Fig. 5 for the twenty-eight ^90^Y treatments, where administered activities ranged from 0.9 to 7.9 GBq with an average value of 3.7 GBq. Seven patients had two administrations of Y-90, and the absorbed dose is the combined absorbed dose from the two treatments. In all but one case (#38), the absorbed dose corresponding to planar imaging was higher than the absorbed dose corresponding to SPECT/CT with AC and SC. The average lung absorbed dose from planar imaging was 9.2 Gy (range from 1.3 to 29.4 Gy) and from SPECT/CT was 3.2 Gy (range from 0.4 to 13.3 Gy). There were no reported cases of subacute lung toxicity; however, our follow-up time is too short (range from 3 to 10 months) to assess late phase toxicity.

#### Impact of time delay between ^99m^Tc-MAA injection and scan

**Fig. 6:**
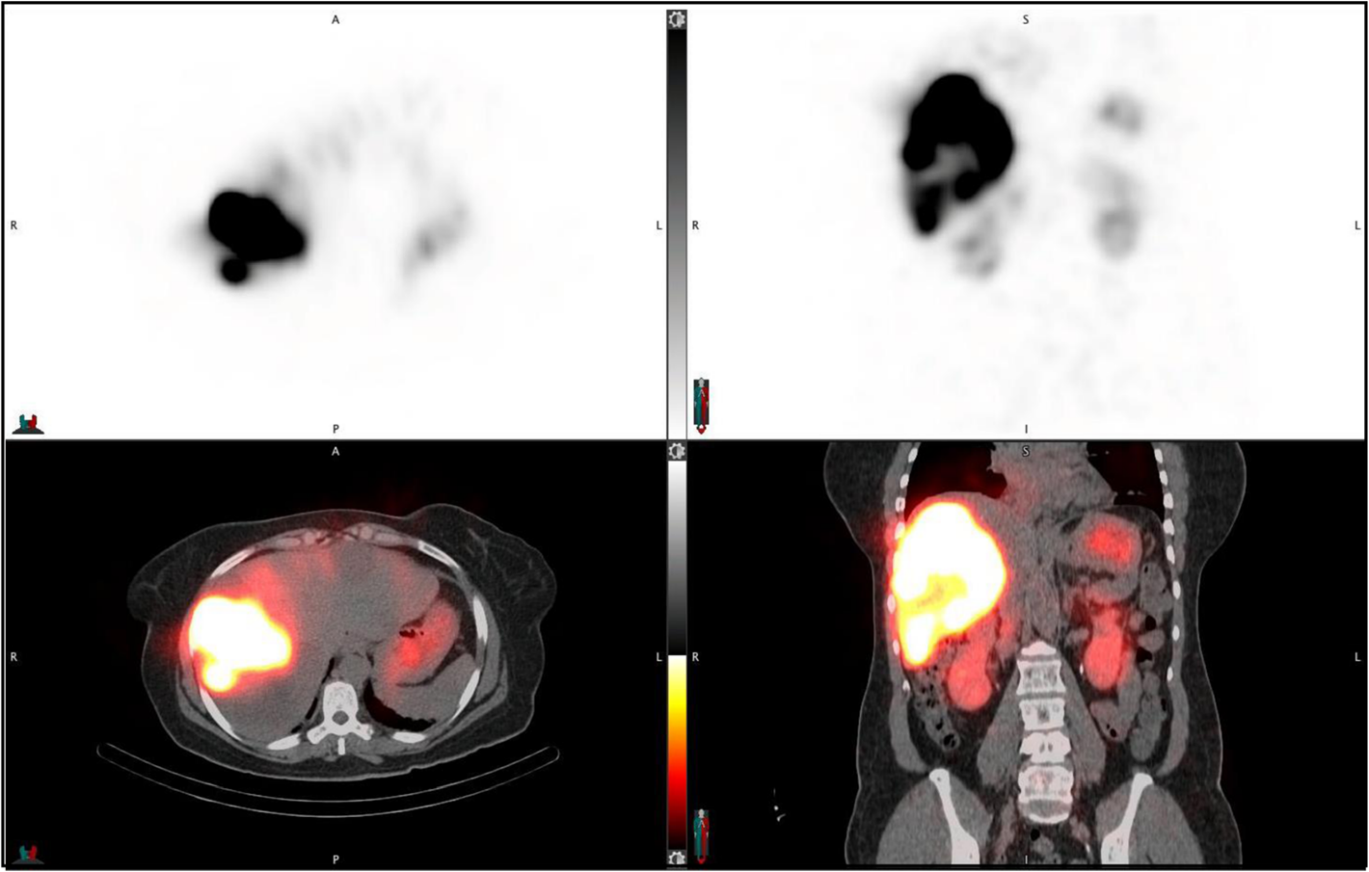
^99m^Tc-MAA SPECT/CT images corresponding to a patient where the time between injection and imaging was 176 min demonstrating gastric mucosal and renal cortical extra-hepatic uptake

The elapsed time between ^99m^Tc-MAA administration and scanning was on average 74 min (range from 25 to 193). No correlation was found between the time delay and the planar LSF (*R*^2^ = 0.04) or SPECT/CT LSF (*R*^2^ = 0.06). Results of the image review for extra hepatic uptake are summarized in Table 4, categorized based on the time delay. Locations of extra hepatic uptake observed included renal, gastric, lung, urinal, and thyroid. An example case demonstrating definitive renal and gastric uptake when imaged after a 176 min delay is shown in Fig. 6.

**Table 4:** Number of patients showing extra hepatic activity on image review categorized based on the delay between the ^99m^Tc-MAA injection and scan

Time delay (min)	Number patients in category	Number with definitive extra hepatic activity visible
0–45	11	0
45–90	18	5
90–135	8	5
135+	3	2

#### Lung shunt from post-therapy ^90^Y PET/CT

**Fig. 7:**
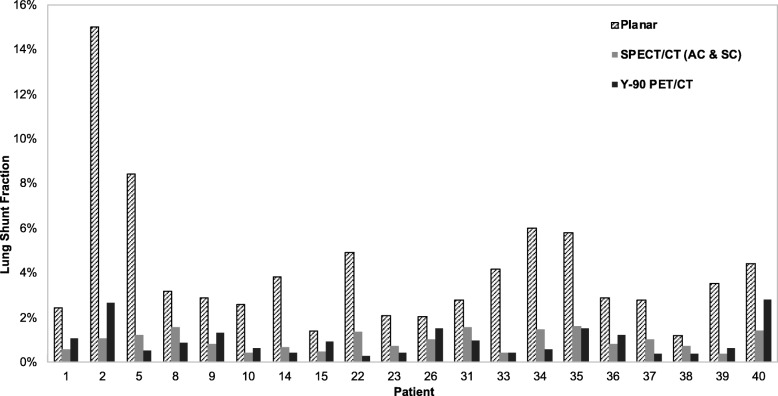
Comparison of pre-therapy ^99m^Tc-MAA and post-therapy ^90^Y PET/CT-based lung shunt estimates (*N* = 20)

As with SPECT/CT, the workflow enabled automatic determination of PET/CT-based LSFs with only minimal user intervention to select one of the pre-set threshold levels to best define the liver contour. The selected levels (number of patients in parenthesis) were 0.5% (4), 1% (3), 2% (10), or 5% (3). For the 20 patients with post-therapy imaging, the ^90^Y PET/CT-based LSFs with and without the exclusion region are given in Table 3. Although 7/20 patients had two Y-90 treatment, the PET results presented here correspond to the first treatment. Post-therapy imaging-based LSFs are compared with pre-therapy imaging-based estimates in Fig. 7 for all patients and show the better agreement when SPECT/CT is used instead of planar imaging. The results summarized in Table 5 show no statistically significant difference between the ^90^Y PET/CT and ^99m^Tc-MAA SPECT/CT-based estimates. However, there were significant mean differences when comparing ^90^Y PET/CT to planar imaging. When compared with ^90^Y PET/CT, 3/20 patients had an absolute difference in LSF that was > 5% with planar imaging while no patients had a difference > 5% with SPECT/CT.

**Table 5:** Comparison of Y-90 PET/CT vs. ^99m^Tc-MAA imaging-based LSFs for the 20 patients that had post-therapy imaging

	LSF, mean (range)	*P* ^*^	Maximum absolute difference in LSF (%)
^99m^Tc-MAA planar imaging	4.1% (1.2–15.0)	0.0002	12.3
^99m^Tc-MAA SPECT/CT (AC and SC)	1.0% (0.4–1.6)	0.968	1.6
^90^Y PET/CT	1.0% (0.3–2.8)		

*P value for significance of difference between mean LSF from indicated method and Y-90 PET/CT

## Discussion

In this study, we investigated the potential value of ^99m^Tc-MAA SPECT/CT imaging compared with planar imaging for lung shunt calculation prior to TARE. The phantom study performed under clinically realistic conditions demonstrated that improved accuracy in LSF estimation (within 13% underestimation of true value) can be achieved using SPECT/CT with AC and SC compared with planar imaging (up to 44% overestimation). Compared with SPECT/CT with AC only, adding SC improved the LSF estimate for the case with the lowest LSF only. For the other cases, the underestimation in LSF with SC is potentially due to inaccuracies in the energy window-based SC. In a previous Monte Carlo simulation study [17], we demonstrated that the TEW method overestimates scatter in the lung region, thereby leading to an underestimation in the reconstructed counts. Other more sophisticated model/Monte Carlo-based methods have been shown to be superior to energy window-based SC [10, 18, 19], but are not always available with clinic systems. For the planar calculation, using the anterior view only for the liver counts and posterior view only for the lung counts, as in our patient studies, led to higher accuracy than using the geometric mean method. The geometric mean method results in higher LSFs because the liver counts go down substantially when the posterior view is included in the calculation while the lung counts are less sensitive to the inclusion of the anterior view. Both planar and SPECT/CT-based estimates had high precision except for the 0% lung shunt case where applying AC and SC to SPECT/CT resulted in high variability due to the very low counts in the lung region (Table 2). Clinically, this will have no impact because of the 0% lung shunt value. The phantom study did not assess respiratory motion effects, or the variability associated with manual segmentation of the liver and lung regions in the planar scans, which are relevant factors in clinical studies.

In patients, in addition to the overestimation in LSF due to lower attenuation in lung tissue relative to liver tissue, resolution effects and respiratory motion artificially increases the value due to liver counts spilling over to the inferior part of the lung. Hence, instead of using the entire lung volume, an exclusion area was used with SPECT/CT and PET/CT, but such a correction is difficult to apply in planar imaging due to the lack of anatomical information. In one case, the SPECT/CT-based LSF was as high as 12.6% without the exclusion region, but reduced to only 2.2% with the exclusion region. The patient results show the same trend as in the phantom study with the SPECT/CT-based LSF decreasing with AC and decreasing further with SC. As in the phantom study, in general, the impact of SC was less than the impact of AC, except at low LSFs. However, we believe SC should be performed when available because improved accuracy was shown in the phantom study at zero LSF and can also improve visibility/contrast of extra-hepatic deposition. The difference between LSFs corresponding to planar imaging and SPECT/CT was generally much higher in patients than in the phantom, which can be attributed to the fact that the phantom was not impacted by motion effects and operator variability in defining regions both of which can significantly impact the planar calculation in patients. The planar-based estimate can have considerable variability associated with the manual segmentation of patient liver and lung regions, which is operator-dependent. The operator variability can be all but eliminated with SPECT/CT-based automatic contouring tools, such as those used in the current workflow. Additionally, in patients, the planar LSFs can be affected by extra-hepatic uptake due to breakdown of ^99m^Tc-MAA over time because unlike with SPECT, planar imaging does not differentiate between activity originating in the source region and originating in overlying and underlying tissue. There can also be scattering off of the structures that are overlying or underlying the source region (for example, liver counts scattering off of breast tissue that is anterior to the lung will contribute to the planar lung counts, but not to the SPECT VOI counts), which can be mitigated by a simple energy window-based scatter correction. In our study, only photopeak window data was available for the clinic planar acquisitions; hence, scatter correction could not be performed. The relative impact of the various effects can be estimated by comparing the LSFs in Tables 2 and 3 with and without the different corrections and the exclusion zone. Based on these results, the use of attenuation correction and the exclusion zone had the biggest impact on SPECT LSF. We were not able to assess the impact of SPECT resolution recovery because all reconstructions included this (no option to turn resolution recovery off in Flash 3D).

Our patient and phantom results for planar vs. SPECT/CT-based LSF estimates are consistent with the recent report of Dittman et al. [7]. For the phantom study, they reported 40% overestimation in LSF with planar imaging and 5% accuracy with SPECT/CT. As in our study, for patients, they reported a much higher difference between planar and SPECT/CT-based LSF estimates than in the phantom study (for patients, their planar to SPECT/CT LSF ratio was on average 3.6, while ours was 4.3). They reported a mean LSF of 8.3 (range from 3.4 to 32.3%) for planar imaging and 2.9% (range from 0.8 to 15.7%) for quantitative SPECT/CT. The corresponding values from our study are 5.4% (range from 1.2 to 15.7%) and 1.5% (range from 0.4 to 6.0%). Their correlation between planar and SPECT/CT-based LSF estimates (*R*^2^ = 0.83) was higher than the correlation we observed (*R*^2^ = 0.46 in Fig. 4), potentially due to using a more systematic approach for planar ROI definition in their prospective study compared with our retrospective use of clinically defined ROIs. Additionally, their planar estimate was based on the geometric mean approach while we used the anterior liver and posterior lung. Their study did not include post-therapy LSF estimates for comparison with ours.

The overestimation in LSF translates to an overestimation in absorbed dose. In one subject, the SPECT/CT-based absorbed dose to the lung was 6.5 Gy (lung shunt 2.5%) while that based on planar imaging was as high as 29.4 Gy (lung shunt 13.6%), which is close to the recommended upper limit for treatment eligibility. The limitation of assuming a “standard” lung mass of 1 kg for all patients in the planar absorbed dose calculation was evident from the individual CT volume-based lung mass for this cohort (average 0.87 kg, range from 0.45 to 1.43) used for the SPECT/CT-based calculation. The maximum tolerable dose to the lung from Y-90 microspheres is not well established. Historically, significant toxicity was associated with a total lung dose of 25 Gy in external beam radiation therapy [20], but this is likely not applicable to TARE due to the non-uniform dose distribution of microspheres. The 30 Gy upper limit recommendation of the manufacturer is based on radiation pneumonitis in two clinical reports where lung absorbed dose was calculated from ^99m^Tc-MAA planar imaging-based LSFs [3, 4]. The relatively poor correlation observed in the present study between planar and SPECT/CT-based LSFs and absorbed doses (Figs. 4 and 5) makes it difficult to translate these past planar imaging-based findings to SPECT/CT-based limits. Obtaining accurate LSFs and lung tolerability dose estimates from SPECT/CT-based calculation could allow individual adjusted doses that improve therapeutic outcome while minimizing pulmonary fibrosis.

There was no statistically significant difference between mean LSF estimates from ^90^Y imaging and estimates based on pre-therapy ^99m^Tc-MAA SPECT/CT with AC and SC. As evident in Fig. 7, the absolute difference between Y-90 PET and ^99m^Tc-MAA SPECT/CT-based estimates are small and will not be clinically important at these low LSFs (the largest difference is for patient 2, where the two LSFs are 2.6 and 1.1%, which translates to lung absorbed doses of 3.6 and 1.5 Gy, respectively). For planar imaging, the differences are larger (the largest difference is for patient 2, where the two LSFs are 2.6 and 12.3%, which translates to lung absorbed doses of 3.6 and 15.6 Gy, respectively). The observations of the current study need to be tested with a larger patient cohort including patients with higher LSFs, which can be difficult because high lung shunting is not typical, and the rare cases may be excluded from treatment. A past study with 14 patients reported that ^99m^Tc-MAA SPECT/CT imaging does not accurately predict lung absorbed doses after 166-Ho TARE [21]. A potential explanation for the disparity was the difference in ^99m^Tc-MAA and Ho-166 microsphere distributions due to differences in stability and size of the MAA particles and the microspheres, which is also a concern with Y-90 microspheres.

There was no correlation between ^99m^Tc-MAA-based LSFs and the time delay between injection and scanning. However, in the current study, most of the patients (29/40) were scanned within 90 min of ^99m^Tc-MAA administration (Table 4). A study of four patients who underwent repeat planar whole-body imaging up to 5 h after ^99m^Tc-MAA administration reported marked increase in lung shunt with time (mean lung shunt was 9.3, 14.7, and 22.1% for time intervals of < 1, 1–4, and > 4 h) [13]. In visual assessment of our patient cohort, extra hepatic uptake tended to be more likely in patients with a longer delay between MAA administration and scanning (Table 4). This can be attributed to the breakdown of ^99m^Tc-MAA over time followed by localization of products in the gastric and renal cortex, which warrant early imaging to improve image quality by minimizing this background activity.

A limitation of the phantom study was that it did not include respiratory motion, which prevented evaluation of the effect of breathing and validation of the use of the lung exclusion region. Motor controlled movable nuclear medicine phantoms have been designed to investigate motion effects [22] and can be considered in a future study. Other limitations of the study include the heterogeneous patient population and the small sample size, especially for post-therapy imaging, where only 20 studies were available as this was not part of the routine clinical procedure.

## Conclusions

A phantom study with clinically realistic uptake patterns for the liver and lung demonstrated the improved accuracy of lung shunt estimates based on SPECT/CT with attenuation and scatter corrections over estimates from planar imaging. The patient studies demonstrated the same trend observed in the phantom study with substantially higher lung shunt estimates from planar imaging compared with those determined by SPECT/CT with corrections. There was no statistically significant difference between LSFs from post-therapy ^90^Y PET/CT and pre-therapy ^99m^Tc-MAA SPECT/CT with attenuation and scatter corrections. There was no correlation between lung shunt estimates and the time delay between MAA injection and scanning, although on visual assessment, there was evidence of increased off-target uptake in extra-hepatic organs with time. In summary, the improved accuracy demonstrated by the phantom study, the agreement with ^90^Y PET/CT and practicality of using auto-contouring for liver/lung definition suggests that ^99m^Tc-MAA SPECT/CT should be used for lung shunt estimation in TARE.

## Abbreviations

2D: Two dimensional
3D: Three dimensional
^90^Y: Yttrium-90
^99m^Tc: Technetium-99m
AC: Attenuation correction
CT: Computed tomography
FOV: Field of view
HCC: Hepatocellular carcinoma
LSF: Lung shunt fraction
MAA: Macroaggregated albumin
OSEM: Ordered subset expectation maximization
PET: Positron emission tomography
R: Pearson’s correlation coefficient
ROI: Region of interest
SC: Scatter correction
SPECT: Single Photon Emission Computed Tomography
TARE: Transarterial radioembolization
VOI: Volume of interest
